# Synergistic Therapies as a Promising Option for the Treatment of Antibiotic-Resistant *Helicobacter pylori*

**DOI:** 10.3390/antibiotics9100658

**Published:** 2020-09-30

**Authors:** Paweł Krzyżek, Emil Paluch, Grażyna Gościniak

**Affiliations:** Department of Microbiology, Wroclaw Medical University, 50-368 Wroclaw, Poland; emil.paluch@umed.wroc.pl (E.P.); grazyna.gosciniak@umed.wroc.pl (G.G.)

**Keywords:** *Helicobacter pylori*, checkerboard assay, synergism, drug interaction, drug combination

## Abstract

*Helicobacter pylori* is a Gram-negative bacterium responsible for the development of gastric diseases. The issue of spreading antibiotic resistance of *H. pylori* and its limited therapeutic options is an important topic in modern gastroenterology. This phenomenon is greatly associated with a very narrow range of antibiotics used in standard therapies and, as a consequence, an alarmingly high detection of multidrug-resistant *H. pylori* strains. For this reason, scientists are increasingly focused on the search for new substances that will not only exhibit antibacterial effect against *H. pylori*, but also potentiate the activity of antibiotics. The aim of the current review is to present scientific reports showing newly discovered or repurposed compounds with an ability to enhance the antimicrobial activity of classically used antibiotics against *H. pylori*. To gain a broader context in their future application in therapies of *H. pylori* infections, their antimicrobial properties, such as minimal inhibitory concentrations and minimal bactericidal concentrations, dose- and time-dependent mode of action, and, if characterized, anti-biofilm and/or in vivo activity are further described. The authors of this review hope that this article will encourage the scientific community to expand research on the important issue of synergistic therapies in the context of combating *H. pylori* infections.

## 1. Introduction

*Helicobacter pylori* is a Gram-negative bacterium that belongs to Epsilonproteobacteria [1]. This microorganism is able to colonize and survive in stomach, which may lead to the development of physiological and morphological changes in this organ [2,3]. The appearance of gastric diseases is driven by the presence of a wide array of *H. pylori* virulence determinants, including toxins, enzymes, and adhesins [4,5]. All of them are responsible for countering the host’s defense mechanisms and establishing long-term colonization. Because of this fact, researchers and clinicians around the world recommend that infections produced by *H. pylori* should always be treated [6,7]. Nowadays, however, an alarmingly high detection of multidrug-resistant (MDR) *H. pylori* strains is observed [8]. Factors associated with the appearance of MDR strains include antibiotic abuse, therapeutic failures, and phenotypical mechanisms promoting resistance/tolerance of these bacteria to antimicrobial substances (efflux pumps expression, biofilm formation, or morphological transformation) [9,10,11,12]. High prevalence, carcinogenic potential, and ability of dynamic resistance spreading constituted the premise for including *H. pylori* in the list of twelve priority pathogens and the need to search for new methods eradicating this microorganism [13].

Currently, there is no progress in development of vaccines directed against *H. pylori* infections, with most studies being in a preclinical phase [14,15]. The proposed preparations include DNA-based, live vector, and ghost vaccines, while none of them proved to be effective enough. Probiotics, although helpful in alleviating negative symptoms associated with *H. pylori* antibiotic therapies, when used as a monotherapy have a low curability rate [16]. This has contributed to the situation in which antibiotics are the only accepted form of combating *H. pylori*, which in turn conditions the growing resistance of this microorganism to these medications [17]. The aforementioned phenomenon is exacerbated additionally by the narrow spectrum of antibiotics that are used to treat *H. pylori* infections (amoxicillin [AMX], clarithromycin [CLR], tetracycline [TET], metronidazole [MTZ], levofloxacin [LEV], and rifabutin [RIF]) [6,7,18]. For this reason, scientists are increasingly focused on the search for new substances that will not only exhibit antibacterial effect against *H. pylori*, but also enhance the activity of other antibiotics.

Synergistic therapies consist of the use of a combination of two or more substances, which when used together directly or indirectly increase the effectiveness of the treatment [19,20,21]. Such therapies are aimed at increasing the spectrum of empirical therapies (during which the pathogen or its resistance is unknown), reducing the possibility of developing resistance, and/or reducing the toxicity of substances used [20,22]. The interactions between compounds are classically determined in microbiological laboratories by establishing a fractional inhibitory concentration index (FICI). Values lower than 0.5 are assumed to be synergistic, results ranging from 0.5–1.0 give additivity (partial synergism), values higher than 1.0 but lower than 4.0 show neutral interactions, while FICI of >4.0 indicates antagonism [20].

Demonstration of synergistic interactions in vitro between the various antibiotics [23,24] or between antibiotics and proton pump inhibitors (PPIs) [24,25,26], currently used in the *H. pylori* therapy, has already taken place in the 1990s and their common application in multidrug therapies is now standard in the treatment of *H. pylori*. Also for bismuth salts, over 30 years ago the synergy of activity with antibiotics against *H. pylori* was determined [27,28], which over the years and taking into account the clinical and epidemiological data allowed for the introduction and widespread use of this therapy around the world [29]. Given all of the above, the importance of in vitro studies aimed at developing new *H. pylori* eradication therapies taking into account the positive interactions between their components, which are then supported by clinical trials, seems to be of a paramount importance.

The aim of the current review is to present scientific reports showing newly discovered or repurposed compounds with an ability to enhance the antimicrobial activity of classically used antibiotics against *H. pylori*. To gain a broader context in their future application in therapies of *H. pylori* infections, their antimicrobial properties, such as minimal inhibitory concentrations and minimal bactericidal concentrations (MICs/MBCs), dose- and time-dependent mode of action, and, if characterized, anti-biofilm and/or in vivo activity are further described.

## 2. Review Strategy and Discussion

The core of the current review consisted of original papers searched for by using the Scopus database and the following terms: “*Helicobacter pylori*” OR “*H. pylori*” AND “synergism” OR “synergistic” OR “drug interaction.” The papers published in the period 2010–2020 (up to July 31) were taken into account. In this way, 259 results were obtained, all of which were subjected to the next screening phase. Works that did not meet one or more of the following criteria were rejected: (1) article written in a language other than English; (2) research on *H. pylori* and antimicrobial substances were not carried out; (3) performing of in vivo tests without taking into account the in vitro activity tests; (4) lack of checkerboard tests as a method of the compounds interaction assessment (the most commonly used method of detecting the presence of interactions between substances); and (5) determining the interaction between two newly discovered substances, e.g., components of plant extracts (only the interactions between the newly discovered substance and the classically used antibiotic(s) were taken into account). The application of the above criteria contributed to the receipt of 14 articles, which were then used in the discussion and summary of the presented results.

### 2.1. Chemically Synthesized Inorganic Compounds

#### Silver Ultra-Nanoclusters

Bionanotechnology has undergone strong development in recent years (Table 1) [30,31,32,33,34]. The attention of many researchers was drawn to the broad antimicrobial activity of nanoparticles and the possibility of using them in various sectors of the economy, including production of bionanomaterials, antimicrobial preparations, dressings, or cover surfaces. The properties of nanoparticles largely depend on their shape, size, and morphology. Ultra-nanoclusters are structures with very low dimensions (<5 nm) and low toxicity toward mammalian cells, which is related to their high stability and unnecessity to use chemical stabilizers. Because of the promising antimicrobial properties and the multi-target mechanism of action (cell membrane, genetic material, protein synthesis, and energy production) ultra-nanoclusters are currently investigated against various gastrointestinal pathogens, including *H. pylori*.

**Figure 1:**
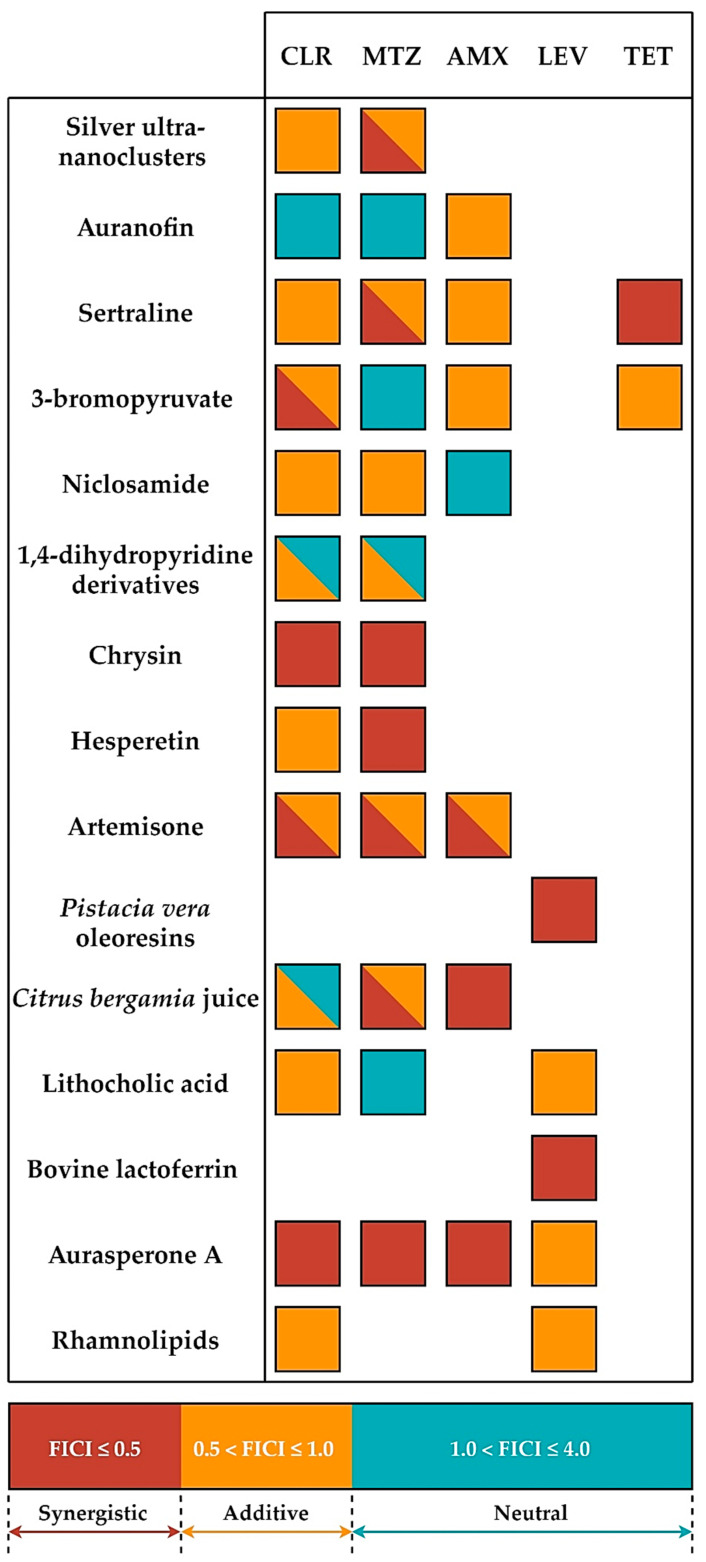
Holistic presentation of interactions between new non-antibiotic compounds and classically used antibiotics against *H. pylori*.

**Figure 2:**
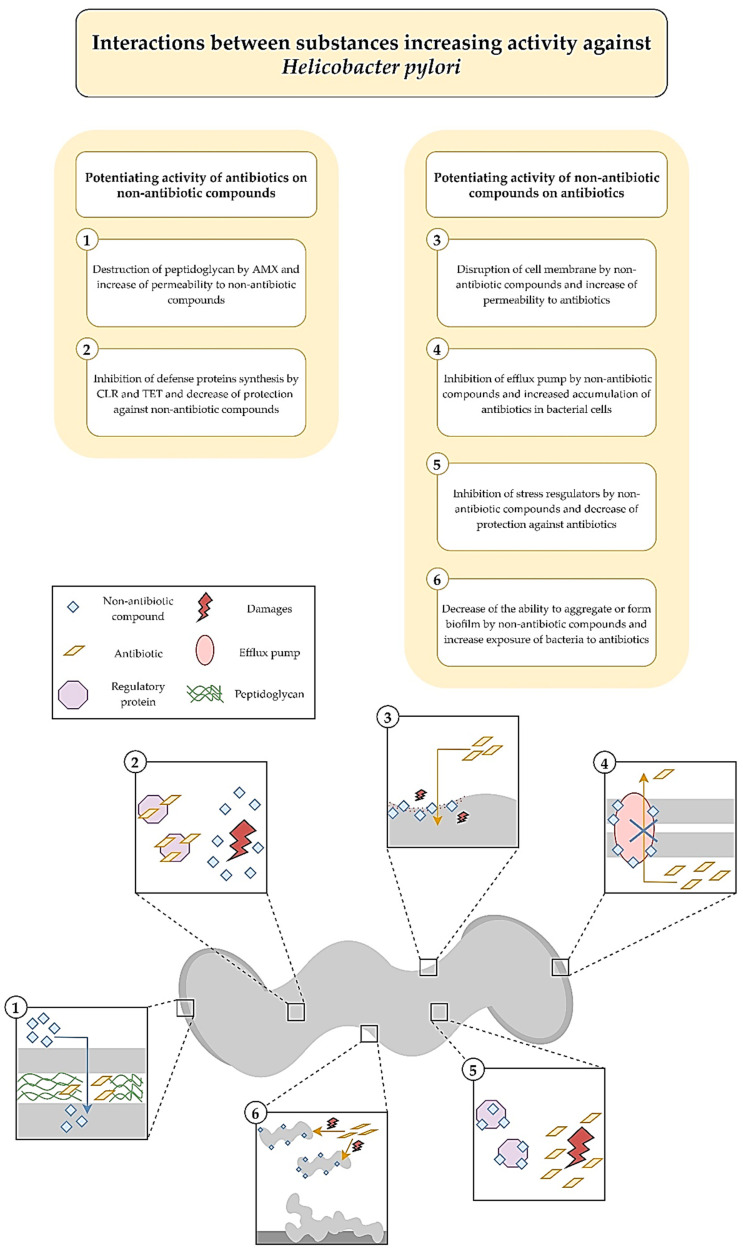
Potential molecular mechanisms conditioning the positive interactions between new non-antibiotic compounds and classically used antibiotics against *H. pylori*.

In a study by Grande et al. an activity of silver ultra-nanoclusters against planktonic and biofilm *H. pylori* forms was verified [34]. It was shown that MICs/MBCs and minimal biofilm eradicating concentrations (MBECs) counted for 0.16–0.33 and 0.64–1.28 µg/mL, respectively. When determining the activity of silver ultra-nanoclusters over time, the existence of a strong bactericidal activity against planktonic forms of *H. pylori* was proved, because MICs contributed to a total decrease in the viability after a 24-h incubation. The one-day exposure to MBECs had a strain-dependent effect ranging from highly bactericidal (a complete loss of the viability) to bacteriostatic (a 1-log reduction). The anti-biofilm activity was further confirmed by observations with fluorescence microscopy, showing an increase in the amount of destroyed LIVE/DEAD-stained cells (presence of red fluorescence). In the study establishing the interaction of silver ultra-nanoclusters with antibiotics, an additive interaction with CLR (FICI = 0.51) and an additive (FICI = 0.55) or synergistic (FICI = 0.42) interaction with MTZ were noticed (Figure 1). The authors of the article hypothesized about the possibility of silver ultra-nanoclusters to induce the detachment of bacteria from the surface, which reduced their ability to produce biofilms and increased sensitivity to antibiotics (Figure 2).

### 2.2. Chemically Synthesized Organic Compounds

#### 2.2.1. Auranofin

Auranofin, a gold phosphate derivative, because of its immunomodulatory properties (an ability of reducing inflammations and cytotoxicity of immune cells) and low harmfulness to human body has been approved by the Food and Drug Administration agency (FDA) in the treatment of rheumatoid arthritis (Table 1) [35,36,37,38,39]. Subsequent research on auranofin indicated not only the usefulness of this compound in the treatment of Alzheimer’s and Parkinson’s disease, but also its antimicrobial activity, including bacteria, fungi, parasites, and viruses. The molecular mechanism against aforementioned group of microorganisms consists of the potential to inhibit the synthesis of many macromolecules crucial for the microbial functioning (DNA, proteins, and lipids). However, the main mechanism of activity is associated with the disruption of the oxido-reductive systems functioning and generation of oxidative stress.

The group led by Owings et al. decided to verify an activity of auranofin against planktonic *H. pylori* forms, showing the strong bactericidal effect of this compound [39]. The MICs were identical to MBCs with values of both being 1.2 µM (0.82 µg/mL). Moreover, the ability of auranofin to inhibit thioredoxin reductase (TrxR), an enzyme responsible for an oxidoreductive homeostasis establishment, with a half maximal inhibitory concentration (IC_50_) of 88 nM (0.06 µg/mL) has also been demonstrated. The study of interactions with antibiotics provided an information about the existence of additivity with AMX (FICI = 0.609) and a neutral, albeit close to additivity, interaction with CLR or MTZ (FICI = 1.03) (Figure 1). The authors of this article suggested that AMX may result in an increased auranofin uptake by *H. pylori* cells (Figure 2).

#### 2.2.2. Sertraline

Sertraline is an antidepressant drug that is widely applied in the treatment of anxiety and obsessive-compulsive disorders (Table 1) [40,41,42,43,44,45]. The mechanism of action of this compound is based on the inhibition of serotonin reuptake. The widespread consumption of sertraline has prompted many researchers to determine the effect of this substance on microbial cells. In recent years, a number of studies have been carried out showing significant antiparasitic, antifungal, antiviral, and antibacterial effects. Sertraline is active against various target sites with a capacity to disturb the stability of cell membranes, interfere with production of genetic material and proteins, and slow down the metabolism of microorganisms. The greatest hopes for the use of sertraline in synergistic therapies, on the other hand, are related to the ability to inhibit efflux pumps, proteins determining nonspecific, but often clinically important, resistance to many groups of antibiotics.

Krzyżek et al. in in vitro studies focused on determining an activity of sertraline against planktonic *H. pylori* forms, taking into account spiral and coccoid forms of this bacterium [45]. The MIC values ranged from 2–8 µg/mL, while MBCs were equal to 4–8 µg/mL. Time-killing assays manifested an ability of this compound to completely diminish the viability after 24 h of incubation with 2 × MIC and 4 × MIC, while MIC equal to MBC (but not MIC ≠ MBC) reduced this parameter by >4 logs. This experiment was independently confirmed by fluorescence microscopy and an 8-fold (*H. pylori* Tx30a) or 15-fold (*H. pylori* J99) reduction in mean green/red fluorescence of LIVE/DEAD-stained cells exposed for 24 h on 4 × MIC was found. The bactericidal activity of sertraline was confirmed despite the presence of coccoid *H. pylori* forms. In the last step of the study, sertraline was shown to have a synergistic or additive interaction with all four tested antibiotics, i.e., TET (FICI = 0.375), AMX (FICI = 0.75–1.0), CLR (FICI = 1.0), and MTZ (FICI = 0.5–1.0) (Figure 1). For example, ¼ × MIC of sertraline allowed for an 8-fold reduction in MIC of TET. In addition, the ability of this substance in ½ × MIC to lower concentrations of MTZ (from 256 to 16 µg/mL, FICI = 0.56) was demonstrated for the MTZ-resistant *H. pylori* strain. At this point, the authors suggested about the possibility of sertraline to inhibit efflux pumps and enhance non-selectively the activity of various antibiotics (Figure 2).

#### 2.2.3. 3-Bromopyruvate

3-Bromopyruvate (3-BP) is a halogen analog of pyruvic acid and a molecule structurally related to lactic acid (Table 1) [46,47,48,49]. The potential of the practical use of this compound in medicine is related to its anti-cancer potential. It has been noticed that in relation to cancer cells, 3-BP acts as a “metabolic toxin”, disrupting metabolic processes (mitochondrial respiration, glycolysis, and ATP synthesis), and generating the oxidative stress-related genetic material destruction. In recent years, it has been shown that a similar mechanism of activity takes place in microbial cells. On this basis, it seems that the use of 3-BP in the future may be important for the simultaneous treatment of gastric tumors and *H. pylori* infections.

In another study by Krzyżek et al. an activity of 3-bromopyruvate (3-BP) against planktonic *H. pylori* forms present in both spiral and spherical forms was checked [49]. The MIC values were determined to be 32–128 µg/mL, while MBCs for all strains were 128 µg/mL. The observation of the antibacterial effect of 3-BP over time showed a fast and strong action of this compound, as concentrations equal to 4 × MIC, 2 × MIC, and MIC contributed to a complete reduction of the *H. pylori* viability after 2 h, 4 h, and 6 h, respectively. The analysis of mean green/red fluorescence ratio of cells confirmed the earlier results and showed a several-fold decrease in this parameter compared to the untreated samples. Here, similarly to sertraline, the bactericidal activity was noticed for both morphological forms of this bacterium. The checkerboard tests demonstrated synergism/additivity with CLR (FICI = 0.5–0.75, in both cases allowing a four-fold MIC reduction of CLR) and additivity with AMX or TET (both FICI = 0.75–1.0) (Figure 1). The authors of the article pointed to the ability of AMX to disrupt the integrity of cell membranes and an integrity-dependent increase of 3-BP uptake or the capacity of the tested translation-disrupting antibiotics (CLR and TET) to reduce the adaptability of 3-BP-exposed bacteria as potential mechanisms responsible for these positive interactions (Figure 2).

#### 2.2.4. Niclosamide

Niclosamide was initially developed for its molluscicide (anti-snail) activity (Table 1) [50,51,52,53,54]. Later studies showed, however, an ability of this substance to fight helminth infections, which, combined with their low toxicity to humans, contributed to the approval of niclosamide as an antiparasitic drug by the FDA. Niclosamide is currently studied in the context of the treatment of many different non-infectious diseases (oncogenic, neurological, and circulatory conditions) and those caused by microorganisms. The mechanism of antimicrobial action is mainly based on disturbance of respiratory processes and acidification of the cytoplasm. Additionally, a quorum quenching effect (interfering with communication, the so-called quorum sensing) of niclosamide was noticed.

In a study by Tharmalingam et al. an antimicrobial activity of niclosamide against planktonic *H. pylori* forms was verified, and then confirmed in tests with the use of gastric adenocarcinoma (AGS) cell lines and in vivo studies [54]. For niclosamide, MIC and MBC values were shown to be 0.25 µg/mL and 0.5 µg/mL, respectively. Importantly, the activity of this compound was preserved at acidic pH and the MIC of this compound was unchanged during an one-month resistance generation test. In a time killing assay, it was found that a one-day incubation with 4 × MIC reduced the viability of planktonic and AGS-adhered *H. pylori* cells by 2 logs and 3 logs, respectively. The ability of niclosamide to diminish expression of vacuolating cytotoxin A (*vacA*) and the degree of AGS cells vacuolization was also proven in cell line experiments. In in vivo studies with *Galleria mellonella*, it was demonstrated that niclosamide at a dose of 25 mg/kg contributed to 70% of larvae survival after 5 days of culture, while in control samples all larvae were dead after the second day. In checkerboard assays, an additive interaction of niclosamide with MTZ (FICI = 0.75) or CLR (FICI = 1.0) was observed (Figure 1). Using fluorescence methods, niclosamide has been shown to lower transmembrane pH, reducing the proton-motive force. This mechanism may be associated with a decrease in the functioning of efflux pumps and an intensification of the antibiotics action (Figure 2).

#### 2.2.5. Dihydropyridine Derivatives

Dihydropyridine derivatives are a group of compounds with a structure based on pyridine (Table 1) [55,56,57,58,59,60]. These compounds are used as antihypertensive drugs and their therapeutic action is associated with blockage of calcium channels. In addition, dihydropyridine derivatives are one of the model bioantioxidants, which are related to their properties of scavenging free oxygen radicals and acting as reducing agents. Dihydropyridine derivatives, like other FDA-approved substances discussed previously, have attracted the attention of scientists in recent years because of their action on various groups of microorganisms (bacteria, fungi, parasites, and viruses) and their multi-target mechanism of activity (respiratory processes, DNA replication, protein translation, and ATP synthesis).

In research by Gonzalez et al. an activity of dihydropyridine derivatives against planktonic forms of *H. pylori* was checked and this effect was confirmed in in vivo studies [60]. These compounds were found to inhibit the action of HsrA, an OmpR-like regulator of stress response, by combining in a 1:1 stoichiometric ratio (Figure 2). The MIC and MBC values ranged from 4–32 µg/mL and 4–64 µg/mL, respectively, with nisoldipine having the highest antibacterial activity (MIC = MBC, 4–16 µg/mL). A time-killing assay showed that a 24-h incubation with 2 × MIC completely reduced the viability of *H. pylori*. This effect was especially visible for nisoldipine and nicardipine, both of which caused such an effect after 4 h. In the in vivo study it was observed that the gastric colonization of mice by *H. pylori* was significantly lower after one week of oral treatment with 100 mg/kg/day of nimodipine (9.5-fold reduction in the median lethal dose (LD_50_)) and nitrendipine (25.5-fold decrease in LD_50_) than in the control. Additionally, an interaction of the tested dihydropyridine derivatives was found to be additive with CLR (FICI = 0.56–1.0, for all except nitrendipine) or with MTZ (FICI = 0.62–1.0, for three of the six tested compounds, i.e., nifedipine, nisoldipine, and nimodipine) (Figure 1).

### 2.3. Plant-Derived Organic Compounds and Plant Extracts

#### 2.3.1. Flavonoids: Chrysin and Hesperetin

Flavonoids are very common in the plant kingdom, mainly as their pigments, and as a large group of heterogeneous compounds they perform a variety of functions essential to plant physiology, such as chemoattraction or chemorepulsion of pollinators, regulation of the life cycle, or protection against environmental stressors (Table 1) [61,62,63,64,65,66]. Flavonoids have strong anti-inflammatory and antioxidant properties, which translates into their widespread use in ethnomedicine since the dawn of time. They are also often used for local or systemic disinfection, which is directly related to their antimicrobial activity and mechanism of action involving a variety of target sites, including cell membrane and cell wall, genetic material, and proteins functioning. These features, combined with the anti-inflammatory/antioxidant properties of flavonoids, have led to numerous studies of their use in the *H. pylori* therapy.

In another study by Gonzalez et al. researchers decided this time to search for substances capable of inhibiting the activity of the HsrA regulator of planktonic *H. pylori* forms adopting alternative methods (Figure 2) [66]. In order to achieve this effect, they used bioinformatical modelling of interaction of the aforementioned protein with compounds approved by the FDA. Preliminary analysis showed that the most desired effects would be obtained for flavonoids, which was then proven in experimental studies. For all the flavonoids tested, MICs were equal with MBCs being in the range of 4–64 µg/mL. Chrysin and hesperidin turned out to be the most potent (4–8 µg/mL for both) and therefore these compounds were then tested for their time-dependent activity and interactions with antibiotics. In time-killing assays, a complete decrease in the viability was found after an 8-h exposure of *H. pylori* to 2 × MIC of chrysin or hesperidin. For both substances the interaction was synergistic with MTZ (FICI = 0.125–0.5), and synergistic for chrysin (FICI = 0.375) or additive for hesperidin (FICI = 1.0) with CLR (Figure 1).

#### 2.3.2. Artemisone

Artemisinin is commonly isolated from *Artemisia annua* and is a natural substance consisting of a sesquiterpene lactone structure with an endoperoxide ring (Table 1) [67,68,69,70,71]. This compound has gained widespread use as an anti-parasitic drug targeting *Plasmodium* (an etiological agent of malaria). Since its discovery, various attempts have been made to modify it chemically, which contributed, inter alia, to the formation of artemisone, a second generation semisynthetic artemisinin derivative. Artemisone, compared to its predecessor, has more favorable pharmacokinetic properties covering a longer half-life and better bioavailability. Artemisone has been shown to have broad antimicrobial activity and to act on many key biological functions in microorganisms, such as cell membrane and cell wall synthesis, DNA replication, protein synthesis, and the maintenance of oxido-reductive homeostasis.

The aim of the research group Sisto et al. was to determine the antimicrobial activity of artemisinin derivatives, in particular artemisone, against planktonic *H. pylori* cells and intracellular forms of this bacterium [71]. Artemisinin had the highest antibacterial effect with MIC = MBC (0.25–0.5 µg/mL). Importantly, both of these parameters were unmodified by environmental pH. The time-dependence study manifested the complete decrease in the viability of bacteria after a 48-h treatment with MICs. In cell line experiments, however, the use of up to 16 × MIC of artemisone did not have a significant effect on the viability of internalized *H. pylori* cells. A study of interactions of this compound with antibiotics showed that out of the five *H. pylori* strains artemisone was synergistic with AMX, CLR, and MTZ against three, two, and one strains, respectively (Figure 1). For the double-resistant *H. pylori* strain (CLR- and MTZ-resistant), additivity was demonstrated with all three antibiotics, i.e., AMX (FICI = 0.515), CLR (FICI = 0.53), and MTZ (FICI = 0.625).

#### 2.3.3. *Pistacia vera* Oleoresins

*Pistacia* is a widespread plant genus, especially abundant in the Mediterranean region (Table 1) [72,73,74,75]. Classically, this tree is used as a source of pistachio nuts, which are dried green fruits commonly used in gastronomy. In recent years, however, attention has been paid to another valuable product of pistachios, i.e., oleoresins. Oleoresins are phytocomplexes composed of a mixture of many different classes of phytochemicals, including terpenoids, essential oils, fatty acids, and phenols. An antimicrobial action of pistachio oleoresins focuses mainly on the interaction with cell membranes and interference with microbial metabolism. Since these substances are often used in folk medicine in relieving digestive ailments (e.g., abdominal pain), scientists have recently focused on determining their activity against *H. pylori*.

In the experiments performed by Di Lodovico et al. an activity of oleoresins isolated from *Pistacia vera* against planktonic and biofilm *H. pylori* forms was determined [75]. Obtained results were later confirmed in an in vivo model. Pistachio oleoresins proved to have MICs in the range of 780–3120 µg/mL and these were consistent with MBC values. Their activity was preserved under the acidic pH of the environment. In checkerboard assays, synergism of pistachio oleoresins with LEV was demonstrated against 31 out of 32 tested *H. pylori* strains (FICI = 0.18–0.5), allowing for a 4–8-fold reduction in the concentration of this antibiotic (Figure 1). The potentiating activity of both components was then tested against *H. pylori* biofilms and it was found that fractional inhibitory concentrations of both, when used together, lowered the amount of biofilm by 38–60%. In the last stage, using *Galleria mellonella*, the protective effect of the combination of pistachio oleoresins and LEV was found. The authors suggested that the ability of oleoresins to disrupt cell membrane continuity and induce an increased LEV adsorption by bacteria was most likely responsible for the observed synergism of these compounds (Figure 2).

#### 2.3.4. Citrus bergamia Juice

Citruses are one of the most consumed fruits in the world (Table 1) [76,77,78,79,80,81]. Among citrus fruits, special attention is paid to bergamot, a plant that inhabits typically southern province of Italy. Juices obtained from citruses, which are a type of plant extract, are widely used as part of a healthy, balanced diet. Their popularity is associated with a pleasant, refreshing taste and health-promoting effect. Epidemiological studies have shown that their regular consumption may prove beneficial for cardiovascular and immune systems. This phenomenon correlates with the phytochemical composition of citrus juices, which are rich in various types of flavonoids (e.g., neoerythocin, neohesperidin, and naringin). Experiments determining an antimicrobial activity of bergamot extracts have shown their ability to destroy the stability of cell membranes, induce oxidative stress, and slow down metabolism.

Filocamo et al. undertook to determine a bactericidal activity of *Citrus bergamia* juice against planktonic *H. pylori* forms [81]. The extracts were shown to have a MIC range of 0.625–5 µg/mL (MIC_50_ = 2.5 µg/mL). A time-dependency in the antibacterial action demonstrated complete loss of the viability of *H. pylori* exposed to 10 µg/mL and 20 µg/mL of extracts after 8 h. Checkerboard tests presented synergistic activity of bergamot juice with AMX (FICI = 0.093–0.49, FIC_90_ = 0.308) and MTZ (FICI = 0.093–0.75, FIC_90_ = 0.192), as well as additive or neutral with CLR (FICI = 0.56–1.24, FIC_90_ = 1.06) (Figure 1). At this stage, time-killing assays were also performed and the fastest decrease in the viability of planktonic *H. pylori* forms was confirmed for bacteria exposed to the combination of bergamot juice and AMX. The authors speculated that synergism of these components was caused by the capacity of biologically active substances of bergamot juice to disturb the stability of bacterial cell membranes and stimulate a disruption-dependent increase of antibiotics uptake (Figure 2).

### 2.4. Mammal-Derived Organic Compounds

#### 2.4.1. Lithocholic Acid

Bile is a type of secretion produced naturally by mammals and other vertebrates (Table 1) [82,83,84,85,86,87,88]. This fluid is rich in bile acids, which are aliphatic molecules that are conjugated in the liver with taurine or glycine. The conjugated forms of these compounds, known as bile salts, are transferred to intestines where they are processed by the microflora and converted into secondary fatty acids—lithocholic and deoxycholic acid. These metabolites are reabsorbed into the liver from where they can be reused. Bile acids act as detergents through their ability to emulsify lipids and as factors controlling cation homeostasis. In addition to the participation of bile acids in digestive processes, recently attention has also been paid to their immunomodulatory and antimicrobial properties against many pathogens of the digestive system.

The group directed by Gonzalez et al. used bioinformatics methods again to search within the database of FDA-approved compounds and find a substance inhibiting the activity of the ArsR regulator, which is crucial for the adaptation to the acidic environment and colonization of the stomach (Figure 2) [88]. Experiments were performed against planktonic *H. pylori* forms. Among these compounds, lithocholic acid belonging to the group of secondary bile acids proved to be able to inhibit ArsR (interacting in a stoichiometric ratio of 1:1) and to have relatively low MIC and MBC values (both being 32 µg/mL). In the study determining the effect of time on the viability of *H. pylori*, it was shown that concentrations corresponding to 2 × MIC and 4 × MIC completely reduce this parameter after 2 h. Additionally, the possibility for this substance to interact additively with CLR (FICI = 0.53, a 32-fold reduction in the antibiotic concentration) and LEV (FICI = 1.0, a 2-fold decrease in the antibiotic concentration) was observed (Figure 1).

#### 2.4.2. Bovine Lactoferrin

Lactoferrin is an 80 KDa glycoprotein belonging to the transferrin family (Table 1) [89,90,91,92,93]. Lactoferrin is one of the most important proteins being present in all endocrine secretions of mammals. Its presence is detected in the colostrum, milk, saliva, tears, and mucus of digestive and respiratory systems. The key role of this protein in the host is associated with entrapping of free iron ions and preventing multiplication of pathogenic microorganisms. Lactoferrin as a component of the non-specific immune response covers with its antimicrobial capacity many groups of microorganisms (bacteria, fungi, parasites, and viruses). The mechanism of activity is mainly related to the interference with cell wall synthesis, as well as the chelation of iron ions. Additionally, lactoferrin may disturb respiratory processes of microorganisms and induce oxidative stress. Human and bovine lactoferrin have an identical sequence, and therefore that derived from cattle is currently considered in the context of the gastrointestinal infections treatment, where it could be delivered, e.g., by consuming dietary supplements or dairy products.

During studies by Ciccaglione et al. the focus was paid at demonstrating the bactericidal activity of bovine lactoferrin against planktonic *H. pylori* forms and the usefulness of this protein in patients’ therapy [93]. The MIC values were equal to 5–20 mg/mL (MIC_50_ = 10 mg/mL), while MBCs were in the range of 40–160 mg/mL (MBC_50_ = 40 mg/mL). Additionally, it has been noticed that sub-inhibitory concentrations of bovine lactoferrin significantly decrease the mobility of this bacterium. The combination of bovine lactoferrin with LEV reduced concentrations of these components by 4–64-fold and 4–32-fold (FICI = 0.09–0.49), respectively (Figure 1). The observations of in vitro studies were then extended to assess the effectiveness of bovine lactoferrin in the treatment of patients with *H. pylori* infections. It was shown that this protein introduced orally in a dose of 300 mg/kg/day to 10-day therapy with AMX (2 g/day) + LEV (1 g/day) + PPI (80 mg/day) increased the effectiveness of patients’ treatment from 75% (18/24) to 96% (51/53).

### 2.5. Microbes-Derived Organic Compounds

#### 2.5.1. Pyrones and Their Derivatives

Pyrones and their derivatives, including naphtho-γ-pyrones, belong to the group of secondary metabolites produced mainly by environmental filamentous fungi (such as *Aspergillus*, *Alternaria*, and *Cladosporium*) (Table 1) [94,95,96,97]. However, the ability to synthesize these compounds has also been demonstrated for lichens, higher plants, or echinoderms. Naphtho-γ-pyrones are currently researched extensively by biotechnological and pharmaceutical industries for their use as antioxidants, antimutagens, or hormone modulators. For naphtho-γ-pyrones, an antibacterial, antifungal, and antiviral activity have also been demonstrated in recent years. Although the molecular mechanism of these compounds has not been fully understood, naphtho-γ-pyrones have been suggested to be involved in disturbing the synthesis of genetic material and protein translation as well as the interference with production of microbial cell wall.

Gou et al. decided to test an antibacterial effect of pyrones and their derivatives, isolated from the marine-derived strain of *Aspergillus*, against planktonic *H. pylori* forms [97]. Among the 20 compounds obtained, aurasperones had the highest activity with MICs of 4–16 µg/mL. Within them bis-naphtho[2,3-b]pyrones were found to perform better activity than hybrid molecules consisting of naphtho[1,2-b]pyrone and naphtho[2,3-b]pyrone. Moreover, it has been discovered that hydroxyl groups at the C-8 carbon of the lower subunit of these molecules were essential for maintaining the anti-microbial activity against *H. pylori*. For aurasperone A, the studies were extended to check interactions with antibiotics, demonstrating synergism with AMX (FICI = 0.375), CLR (FICI = 0.5), and MTZ (FICI = 0.375), and additivity with LEV (FICI = 0.75) (Figure 1).

#### 2.5.2. Rhamnolipids

Rhamnolipids are representatives of biosurfactants produced by microorganisms, mainly Gram-negative rods of the genus *Pseudomonas*, *Acinetobacter*, and *Burkholderia*, and also Gram-positive *Bacillus* or *Planococcus* (Table 1) [98,99,100,101,102]. Rhamnolipids have for a long time been used in environmental microbiology in bioremediation. The attention of scientists from biomedical fields was attracted by an increasing number of reports indicating the possibility of applying these substances in human therapy. The antimicrobial activity of rhamnolipids has been observed against bacteria, fungi, and enveloped viruses. Despite the fact that biocidal concentrations for microbes are relatively high, very promising modulatory properties against adhesion and biofilm formation are indicated for rhamnolipids. As it is increasingly recognized that *H. pylori* biofilm may play a key role in persistent, difficult-to-treat infections, the use of rhamnolipids may have beneficial therapeutic effects in the treatment of *H. pylori*.

The influence of rhamnolipids on planktonic and biofilm forms of *H. pylori* was tested by a research group of Chen et al. [102]. For the free-swimming forms of this bacterium, the MIC_90_ was 307.2 µg/mL. In checkerboard assays, the additivity of rhamnolipids with CLR (FICI = 0.75) and AMX (FICI = 1.0) was demonstrated (Figure 1). During fluorescence analysis using a LIVE/DEAD staining, a marginal effect of rhamnolipids on the bacterial viability (presence of green fluorescence) was noticed. However, even at sub-inhibitory concentrations the ability of rhamnolipids to significantly reduce biofilm formation was found (¼ × MIC to MIC diminished the amount of biofilm to <20%). This phenomenon prompted the researchers to extend their experiments by determining the activity of rhamnolipids against biofilm forms of *H. pylori*, for which the MBECs were equal to 1228.8 µg/mL (4 × MIC of planktonic forms). Importantly, it was found that rhamnolipids significantly affect the eradication of *H. pylori* biofilms in combinations with antibiotics, especially in the consolidation with AMX (FICI = 0.5, synergism). The authors suggested that the potential of rhamnolipids to micellize lipopolysaccharide and proteins (important biofilms’ components) and modify hydrophobic/hydrophilic properties of bacterial cells may be responsible for reducing biofilm and enhancing the activity of antibiotics (Figure 2).

## 3. Summary

The issue of spreading antibiotic resistance of *H. pylori* and its limited therapeutic options is an important topic in modern gastroenterology. Among the currently used methods of *H. pylori* eradication, a very narrow repertoire of antibiotics is adopted. Treatment modifications are very often restricted only to dosing or replacing one of the antibiotics or proton pump inhibitors with another. It seems that solution to the current impasse may cover the use of new compounds capable of enhancing the activity of currently used antibiotics. This review discusses newly discovered or repurposed substances that have been shown to synergistically or additively interact with antibiotics against *H. pylori*. It is worth noting that in many of these publications the research was limited to in vitro conditions and the interactions of new non-antibiotic compounds were tested in combination with only one or two selected antibiotics. Hence, there is a need for further research into their clinical efficacy and applicability with various antibiotic formulations. The authors of this review, however, hope that this article will encourage the scientific community to expand research on the important issue of synergistic therapies in the context of combating *H. pylori* infections.

## Figures and Tables

**Table 1:** Characterization of non-antibiotic compounds showing a positive antibacterial interaction with classically used antibiotics against *H. pylori*.

Compounds	Structure	Chemical Characteristic	Application	Antimicrobial Spectrum	Mechanism of Action	Reference
Silver ultra-nanoclusters	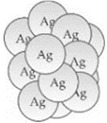	Chemically synthesized inorganic compounds	Components of bionanomaterials, antimicrobial preparations, dressings, and cover surfaces	Bacteria, fungi, parasites, viruses	- Cell membrane damage - DNA replication impairment - Proteins synthesis alteration - ↑ Oxidative stress - ↓ ATP synthesis	[30,31,32,33,34]
Auranofin	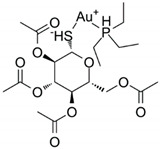	Chemically synthesized organogold compounds	Rheumatoid arthritis drug	Bacteria, fungi, parasites, viruses	- Inhibition of cell wall and DNA synthesis - Proteins and lipids synthesis alteration - ↑ Oxidative stress	[35,36,37,38,39]
Sertraline	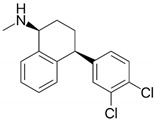	Chemically synthesized organic compounds	Antidepressant drug	Bacteria, fungi, parasites, viruses	- Cell membrane damage - DNA replication impairment - Interference with metabolism - Inhibition of efflux pumps - ↓ Proteins translation - ↓ ATP synthesis	[40,41,42,43,44,45]
3-bromopyruvate	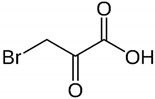	Chemically synthesized organic compounds	Anticancer agent	Bacteria, fungi, parasites	- DNA replication impairment - Interference with metabolism - ↑ Oxidative stress - ↓ Proteins translation - ↓ ATP synthesis	[46,47,48,49]
Niclosamide	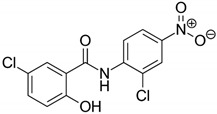	Chemically synthesized organic compounds	Antiparasitic drug	Bacteria, fungi, parasites, viruses	- Disruption of oxidative respiration - Intracellular pH alternations (acidification) - ↓ ATP synthesis - ↓ Quorum sensing	[50,51,52,53,54]
Dihydropyridine derivatives	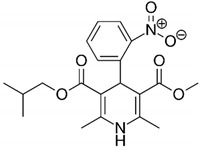 Nisoldipine	Chemically synthesized organic compounds	Hypertension drugs	Bacteria, fungi, parasites, viruses	- Disruption of oxidative respiration - DNA replication impairment - ↓ Proteins translation - ↓ ATP synthesis	[55,56,57,58,59,60]
Flavonoids: chrysin and hesperetin	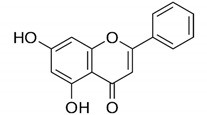 Chrysin 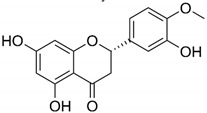 Hesperetin	Plant-derived organic compounds	Antioxidant and anti-inflammatory compounds	Bacteria, fungi, parasites, viruses	- Cell membrane damage - Inhibition of cell wall and DNA synthesis - Inhibition of efflux pumps - ↓ Proteins translation - ↓ ATP synthesis	[61,62,63,64,65,66]
Artemisone	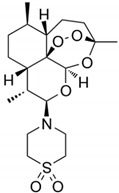	Plant-derived organic compounds	Antiparasitic drug (anti-*Plasmodium* agent)	Bacteria, fungi, parasites, viruses	- Cell wall/membrane damage and synthesis alternations - Interference with metabolism - DNA replication impairment - ↑ Oxidative stress - ↓ Proteins translation - ↓ ATP synthesis	[67,68,69,70,71]
*Pistacia vera* oleoresins	Main components: 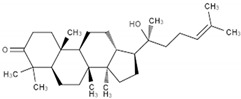 Dipterocarpol 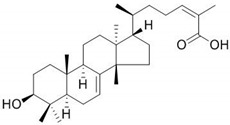 Masticadienonic acid	Plant-derived organic compounds	Used in ethnomedicine for gastrointestinal complaints	Bacteria, fungi, parasites, viruses	- Cell membrane damage - Interference with metabolism - DNA replication impairment - ↓ ATP synthesis	[72,73,74,75]
*Citrus bergamia* juice	Main components: 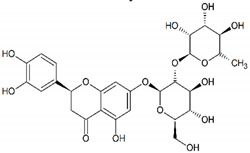 Neoeriocitrin 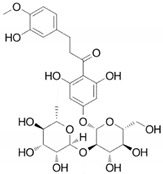 Neohesperidin	Plant extract	Used in ethnomedicine for diabetes and cardiovascular disorders	Bacteria, fungi, viruses	- Cell membrane damage - Interference with metabolism - DNA replication impairment - ↑ Oxidative stress - ↓ ATP synthesis	[76,77,78,79,80,81]
Lithocholic acid (3α-hydroxy-5β-cholan-24-oic acid)	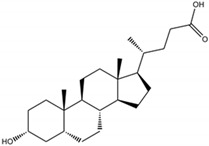	Mammal-derived organic compounds	Natural components of biliary system with digestive, antimicrobial, and immunomodulatory properties	Bacteria, fungi, viruses	- Cell membrane damage - DNA damage and replication impairment - Intracellular pH alternations (acidification)	[82,83,84,85,86,87,88]
Bovine lactoferrin	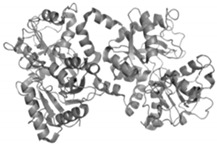	Mammal-derived organic compounds	Natural components of immune system with antimicrobial and immunomodulatory properties	Bacteria, fungi, parasites, viruses	- Cell wall/membrane damage and synthesis alternations - Disruption of oxidative respiration - Iron ions chelation - ↑ Oxidative stress	[89,90,91,92,93]
Pyrones and their derivatives	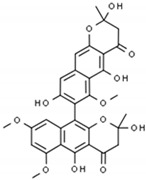 Aurasperone A	Microbe-derived organic compounds	Modulators of hormones	Bacteria, fungi, viruses	- Cell membrane synthesis alternations - DNA replication impairment - ↓ Proteins translation	[94,95,96,97]
Rhamnolipids	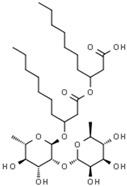	Microbe-derived organic compounds	Antiadhesive and antibiofilm agents	Bacteria, fungi, viruses	- Cell membrane damage and hydrophobicity/hydrophilicity alternations - Destruction of exopolysaccharide by forming micelles - ↑ Oxidative stress - ↓ ATP synthesis	[98,99,100,101,102]
